# Limited T-Cell-Stimulating Effect of Cytochalasin-B-Induced Membrane Vesicles Isolated from Artificial Antigen-Presenting Cells

**DOI:** 10.3390/vaccines10111877

**Published:** 2022-11-07

**Authors:** Yeongwon Kim, Sueon Kim, Cheol-Hwa Hong, You-Seok Hyun, In-Cheol Baek, Tai-Gyu Kim

**Affiliations:** 1 Department of Microbiology, College of Medicine, Catholic University of Korea, Seoul 06591, Korea; 2 Department of Biomedicine & Health Sciences, College of Medicine, Catholic University of Korea, Seoul 06591, Korea; 3 Catholic Hematopoietic Stem Cell Bank, College of Medicine, Catholic University of Korea, Seoul 06591, Korea

**Keywords:** cytochalasin-B-induced membrane vesicles, artificial antigen-presenting cells, Jurkat reporter cells, HLA class I, human cytomegalovirus, pp65

## Abstract

Artificial antigen-presenting cells (aAPCs) that stably express particular HLA and co-stimulatory molecules by gene transfer have been developed to effectively stimulate T cells. To investigate whether cytochalsin-B-induced membrane vesicles derived from aAPCs (AP-CIMVs) have similar antigen-presenting functions as a cell-free system, T cell responses to different types of antigen presentation were measured using Jurkat reporter cells. First, the aggregation of AP-CIMV, which affects the measurement of function, was inhibited by nuclease treatment to produce uniform AP-CIMVs. The Green fluorescent protein (GFP) expression in Jurkat reporter cells was induced in a dose-dependent manner in groups stimulated with anti-CD3 antibody-coated AP-CIMVs and aAPCs, and anti-CD3/CD28 Dynabead. When Jurkat reporter cells expressing specific T cell receptors were stimulated by AP-CIMVs and aAPCs loaded with CMV pp65 peptide, AP-CIMVs showed similar stimulatory effects to that by aAPC. However, when these Jurkat reporter cells were stimulated by aAPCs endogenously expressing CMV pp65 antigen and their AP-CIMVs, the GFP expression rate by AP-CIMVs was 8.4%, which was significantly lower than 53.2% by aAPCs. Although this study showed a limited T-cell-stimulating effect of AP-CIMVs on endogenously processed antigen presentation, these results provide useful information for the development of improved cell-free systems for T cell stimulation in the future.

## 1. Introduction

Extracellular vesicles (EVs) range in size from 50 nm to 5 μm and are heterogeneous, membrane-bound phospholipid vesicles that are actively released by a variety of mammalian cells. [1]. EVs act as paraclinical effectors that deliver biologically active molecules (nucleic acids, proteins, lipids, etc.) to recipient cells to regulate various biological processes and to reform their phenotype, regulate the microenvironment, and induce cellular signal 1 [2]. There are three major subtypes of EVs based on their sizes: exosomes (50–200 nm), microvesicles (100–2000 nm), and apoptotic bodies (1000–5000 nm) [3].

In addition to these naturally occurring EVs, attempts have been made to artificially produce EVs more efficiently through physical or chemical manipulation in order to apply the biological properties of EVs to various fields [4,5]. Cytochalasin B binds and inhibits the actin filament of the cytoskeleton under the cell membrane, which softens and easily produces MVs with sizes of 100–1000 nm by physical shock such as vortexing or hand shaking. Furthermore, cytochalasin-B-induced membrane vesicles (CIMVs) can be simply purified as nanovesicles without cell nuclei [6]. CIMVs of mesenchymal stem cells exhibit angiogenic activity and immunomodulatory efficacy [7]. CIMVs are also able to transfer nucleic acids or anti-cancer drugs to a level similar to that of natural extracellular vesicles [8].

Dendritic cells (DCs) are the gold standard for culturing T cells by expressing major histocompatibility complex (MHC) and co-stimulatory molecules on their surface [9]. The DC exosome (DEX) was developed for application to the cell-free canister vaccine as a substitute for DC and showed a sufficient effect to elicit an anti-cancer impulse response. However, there are difficulties in immunotherapeutic approaches because of the limitations in producing sufficient amounts of DEX [10]. To overcome the limited number of dendritic cells, artificial antigen-presenting cells (aAPCs) have been developed by transfecting cell lines with MHC and co-stimulatory molecules such as CD80, CD83, and CD137L [11,12]. Additionally, aAPC-derived exosomes, such as DC exosomes, have been demonstrated to stimulate antigen-specific T cells [13].

Recently, CIMVs from HeLa cell lines overexpressing recombinant antigens such as CD19, IL-13Ra2, HER2, CD33, and CD123 have been used to stimulate and proliferate T cells transduced with chimeric antigen receptor (CAR) genes in vitro [14]. However, no detailed studies have been conducted on the efficacy of CIMVs in stimulating or proliferating antigen-specific T cells. Therefore, we generated antigen-presenting CIMVs (AP-CIMVs) expressing HLA molecules and co-stimulatory molecules derived from HEK293T-based aAPCs and investigated their immunological properties for T cell stimulation using the NF-κB promoter-based Jurkat reporter cell line. AP-CIMVs expressing HLA molecules present antigens to T cells with properties similar to those of aAPCs.

The ability of AP-CIMVs to stimulate T cells was evaluated using a fluorescent Jurkat cell platform. GFP was designed to be expressed when NF-κB signaling was initiated in Jurkat T cells, which could serve as a platform to confirm T cell activation. NF-κB signaling can be initiated by specific or non-specific CD3 stimulation in both cases [15].

In this study, we produced and purified AP-CIMVs using HEK293T-based aAPC utilizing low non-specific response characteristics and reacted them with a fluorescent Jurkat platform expressing a single TCR via a CMV antigen pulse or natural class I antigen presentation process. The expression level of GFP was measured to compare the CMV antigen-specific and non-specific T-cell-stimulating effects of aAPC and AP-CIMVs.

## 2. Materials and Methods

### 2.1. Cells and Culture Conditions

HEK293T cells (ATCC, Manassas, VA, USA), HEK293T-based aAPCs (HLA null, aAPC, and aAPC-CMV pp65), and Jurkat cells (ATCC, USA) were cultured in RPMI-1640 medium (Lonza, Walkersville, MD, USA) supplemented with 10% fetal bovine serum (Lonza, Walkersville, MD, USA), 1% penicillin–streptomycin (Lonza, Walkersville, MD, USA), and 1% L-glutamine (Lonza, Walkersville, MD, USA). HEK293T-based aAPCs have been previously shown to stably express single HLA class I A*02:01 and co-stimulatory molecules such as CD80, CD83, CD137L, and CD32 [16]. All cells were grown and assayed at 37 °C with 5% atmospheric CO_2_.

### 2.2. Production and Isolation of CIMVs and AP-CIMVs

CIMVs were isolated from native and genetically modified HEK293T cells by treatment with cytochalasin B (Thermo Fisher, Waltham, MA, USA). The purification and isolation method of CIMVs was described in a previous study [7]. HEK293T cells were treated with 0.25% trypsin/EDTA (Lonza, Walkersville, MD, USA) and detaached from the culture plate. The cells were counted and resuspended in serum-free Dulbecco Modified Eagle Medium (DMEM, Lonza, Walkersville, MD, USA) at 1 × 10^6^/mL concentration. Then, 10 mL of the cell suspension was transferred to the T25 plate, and cytochalasin B was added to the plate at 10 μg/mL and mixed. The cells were incubated for 30 min at 37 °C, 5% CO_2_. The cells were vigorously vortexed for 45–60 s and subjected to three subsequent centrifugations (100× *g* for 5 min, 300× *g* for 20 min, and 2000× *g* for 25 min). The pellet was resuspended in 200 μL of PBS and was treated with 1 U/μL of DNase1 (Thermo Fisher, Waltham, MA, USA). The isolated CIMVs were incubated for 15 min at 37 °C to activate the DNase1. AP-CIMVs were washed thrice with PBS and collected from the final pellet. The final pellet of AP-CIMVs was resuspended in 300 μL of fresh PBS or culture medium, depending on the purpose of further experiments. The concentration of CIMVs was measured using BCA (Thermo Fisher, Waltham, MA, USA) or Bradford (Thermo Fisher, Waltham, MA, USA) and stored in a −20 °C freezer.

### 2.3. Characterization of AP-CIMVs

AP-CIMVs were quantified using the Bradford protein assay to measure the total amount of protein in the isolated AP-CIMVs. AP-CIMVs were lysed using Radioimmunoprecipitation assay buffer (RIPA buffer) prior to protein quantitative measurement. Serial dilutions of bovine serum albumin (BSA) were performed for the standard, and the equation of the standard curve was calculated. This equation was used to quantify the levels of isolated AP-CIMVs within the protein. The size of the AP-CIMVs was measured using a Zetasizer Nano Zs (Malvern, Worcestershire, UK), and the expression of co-stimulatory molecules and HLA molecules was measured using flow cytometry.

### 2.4. aAPC Endogenously Expressing CMV pp65 Antigen

Lentiviral transduction was used to establish an endogenous CMV pp65 antigen-expressing 293T-based aAPC. For lentivirus production, 5 × 10^6^ HEK293T cells/10 mL were seeded in T75 flasks. After 24 h, Lipofectamine 2000 reagent was used to co-transfect the lentivirus packaging plasmids (5 μg of pMD2.G and 5 μg of psPAX2; Addgene, Watertown, MA, USA) and 10 μg of a pCDH plasmid (SBI, Palo Alto, CA, USA) encoding CMV-pp65-T2A-tagBFP into HEK293T cells (Invitrogen, Waltham, MA, USA). Then, 48 h after transfection, lentiviral supernatants were collected and passed through 0.45 μM filters. To stably express CMV pp65 via lentiviral transduction, 5 × 10^5^ HEK293T aAPC cells/mL were seeded in 6-well plates. After 24 h, the cells were treated with 1 mL of lentiviral supernatant and 8 μg/mL polybrene. Flow cytometry was used to examine cells 72 h after transduction. Cells were harvested, blue fluorescent protein (BFP)-positive HEK293T cells were sorted, and single cells were seeded in 96-well plates using a BD FACS Aria (BD, Franklin Lakes, NJ, USA). Finally, single cells expressing high levels of CMV pp65 and BFP were cloned and selected.

### 2.5. Flow Cytometry

For analysis, the cells were harvested and incubated with fluorescently labeled anti-human antibodies for 30 min at 4 °C in the dark. Each sample was stained by 1 ul of each antibody at a concentration of 0.2 mg/mL in 100 ul of PBS containing 2% FBS. In flow cytometry, live aAPCs or CD3+ Jurkat cells were gated and recorded in at least 1 × 10^4^ cells to determine the GFP expression rate. The following antibodies were used to detect targeting molecules: anti-CD80-PE (BioLegend, San Diego, CA, USA), anti-CD83-PE (BioLegend, San Diego, CA, USA), anti-CD137L-PE (BioLegend, San Diego, CA, USA), anti-CD-32-FITC (BioLegend, San Diego, CA, USA), anti-CD9-PE (BioLegend, San Diego, CA, USA), anti-CD63-PE (BioLegend, San Diego, CA, USA), anti-CD81-PE (BioLegend, San Diego, CA, USA), anti-CD8α-PerCP-cy5.5 (BioLegend, San Diego, CA, USA), and anti-CD3-BV421 (BioLegend, San Diego, CA, USA). Fluorescence was measured using a BD FACS Canto (BD Biosciences) and analyzed using the FlowJo v10 software (BD Biosciences).

### 2.6. Generation and Stimulation of A*02:01 Specific Binding TCR Reporter Jurkat

CMV pp65 antigen was prepared, and the CMV pp65 epitope TCR was produced and tested in a previous study [17,18]. Purified PCR products (300 ng/μL) were used as templates for in vitro transcription (IVT). Anti-Reverse Cap Analog (ARCA) capping and the synthesis of IVT mRNA was performed with using the MEGAscript T7 Transcription Kit according to the manufacturer’s instructions, and polyadenylation was performed using a Poly(A) Tailing Kit. The final IVT products were purified using the MEGAclear Transcription Clean-Up Kit, according to the manufacturer’s instructions. Then, 1 μM of A*02:01 CMV pp65 IVT mRNA was transfected into aAPCs using the BTX electroporation protocol (400 V with a 500 μs pulse). Next, 1 × 10^5^ Jurkat reporter cells were co-cultured with 1 × 10^4^ aAPCs or AP-CIMVs expressing a single HLA class I allotype by transfection in 200 μL of complete RPMI-1640 medium in flat-bottomed 96-well plates for 18 h. The expression of GFP induced by antigen-specific stimulation via naturally processed CMV pp65 was measured using flow cytometry.

### 2.7. Statistical Analysis

Graphpad Prism 7 (Graphpad software, San Diego, CA, USA) and FlowJo (BD, Franklin Lakes, NJ, USA) were used for statistical analysis and data presentation. The mean (%) ± standard deviation (SD) was determined for all the test groups and control groups. T-tests and one-way ANOVA were used to determine the significance of differences between the treatment and control groups. Statistical significance was set at *p* < 0.05.

## 3. Results

### 3.1. Effect of DNase1 Treatment on Isolated AP-CIMVs

**Figure 1:**
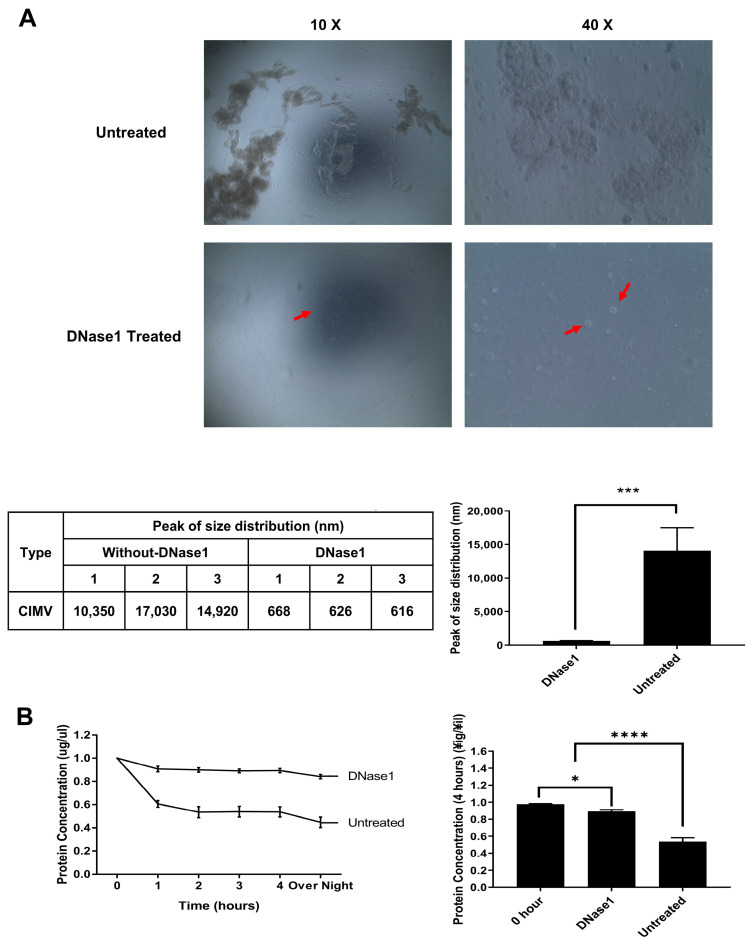
Inhibition of the aggregation of antigen-presenting cytochalasin-B-induced membrane vesicles (AP-CIMVs) generated from artificial antigen-presenting cells (aAPCs) by treatment with DNase1. (**A**) Image of AP-CIMVs’ aggregation without DNase1 and AP-CIMVs treated with DNase1 on Bright-field microscopy after 4 h of incubation (10×, 40× objective). Red points indicate a vesicle in the picture. Size of isolated AP-CIMVs was measured via Dynamic Light Scatter (DLS). Aggregated AP-CIMV’s diameter was measured as average of 14.1 μm, and DNase1-treated AP-CIMV’s was 636.7 nm. (**B**) Comparison of recovery rates of non-aggregate AP-CIMVs after incubation between DNase1-treated and untreated groups. Measurement of protein amount after removal of aggregated vesicles via centrifugation after incubation at 37 °C, 5% CO_2_. The protein amount was measured via Bradford protein assay. The measurement was performed. Error bars display the mean values and ±SD of three replicates. One-way ANOVA or *t*-test was used to obtain the *p* value. * *p* < 0.05, *** *p* < 0.001, **** *p* < 0.0001.

AP-CIMVs isolated using the previously reported general method cause aggregation over time in a cell culture environment. Because such aggregation is not appropriate for investigating the biological effects of AP-CIMVs, a method for stably securing non-aggregated AP-CIMVs was investigated. DNA isolated from cells destroyed by physical force after cytochalasin B treatment was hypothesized to cause aggregation; thus, the effect of DNase1 treatment was measured. The anti-aggregation action of DNase1 could be visually validated by comparing the aggregation of AP-CIMVs according to the treatment with DNase1. Dynamic light scatter (DLS) was used to assess the size of a single AP-CIMV, and statistical values were confirmed by three measurements in both the DNase1-treated and non-treated groups. The average size in the group not treated with DNase1 was 14.1 μm, which was 23.5 times the average 636.7 nm size observed in the DNase1-treated group (Figure 1A).

In order to quantitatively determine the time of aggregation and the ratio of aggregation to non-aggregation AP-CIMVs, freshly isolated AP-CIMVs were incubated at 37 °C at a concentration of 25 μg/mL. Between the first four hours and the final 18 h, harvested AP-CIMVs were centrifuged to remove aggregates, and the protein concentration of the non-aggregated AP-CIMVs in the supernatant was measured using the Bradford protein assay. Aggregation occurred after one hour of incubation, and the ratio of non-aggregated AP-CIMVs decreased to 60% compared to freshly isolated AP-CIMVs. The decreasing trend steadily continued throughout the first four hours, with the ratio at 50% after 18 h. On the other hand, in the case of treatment with DNase1, the ratio remained at 90% after the first four hours. (Figure 1B).

Cytochalasin B was used to form AP-CIMVs on the surface of HEK293T-based APC, and then, serial centrifugation was performed for isolation. DNase1 was used to inhibit the aggregation of AP-CIMVs by releasing DNA from disrupted cells. The final pellet was washed three times with fresh PBS, and the protein concentration of AP-CIMVs was determined using the Bradford protein assay.

### 3.2. Immunological Characteristics of AP-CIMVs

**Figure 2:**
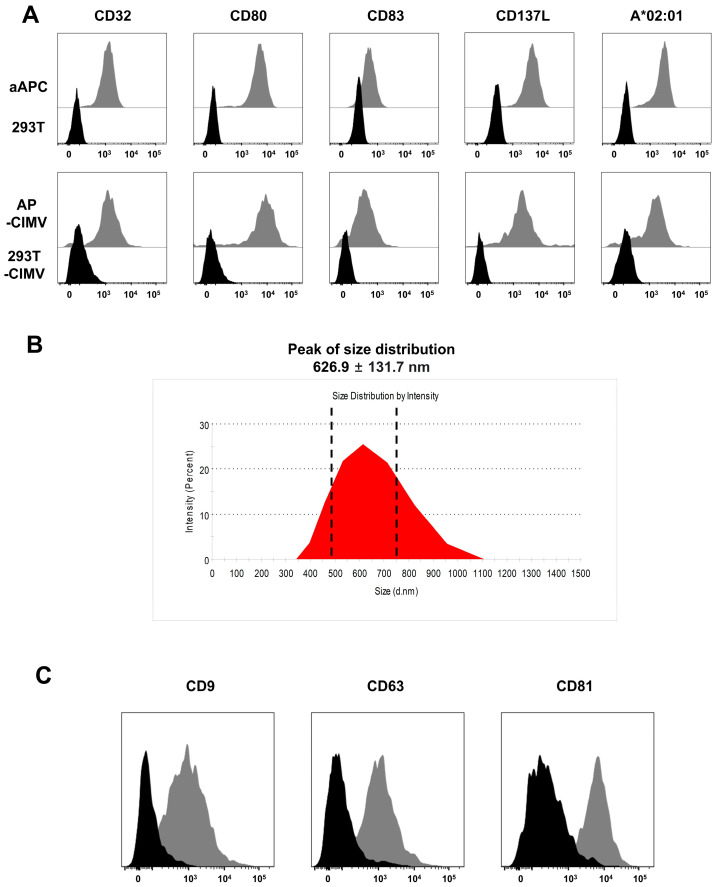
Characterization of AP-CIMVs generated from aAPC expression co-stimulatory molecules and HLA-A*02:01. (**A**) The expression of CD32, co-stimulatory molecules (CD80, CD83, and CD137L) and HLA-A*02:01 on the surface of aAPCs and AP-CIMVs was analyzed via flow cytometry. (**B**) The size of AP-CIMVs was measured via Zetasizer Nano ZS. Majority of the size was confirmed at 626.9 ± 131.7 nm. (**C**) The expression of extracellular vesicle markers (CD9, CD63, and CD81) on the surface of AP-CIMVs was analyzed via flow cytometry. Black histograms indicate isotype control, and gray histograms indicate anti-CD9, CD63, or CD81 stained samples.

The stable expression of the co-stimulatory molecules of CD80, CD83, and CD137L and CD32 capable of binding immunoglobulin and HLA-A*02:01 in HEK293T-based aAPCs was confirmed using flow cytometry. AP-CIMVs generated from these aAPCs also expressed these molecules at similar concentrations (Figure 2A). The size of AP-CIMVs measured by DLS was 626.9 ± 131.7 nm, and the size distribution was 350–1100 nm. They were similar in size to microvesicles, with a size of 200–1000 nm among the extracellular vesicles (Figure 2B). The tetraspanin families CD9, CD63, and CD81, which are characteristic EV indicators, were expressed in AP-CIMVS (Figure 2B). These data indicate that AP-CIMVs isolated from aAPCs have the potential to stimulate T cells by expressing molecules necessary for antigen presentation along with typical microvesicle characteristics.

### 3.3. T Cell Activation by AP-CIMVs Coated with Anti-CD3 Antibody

**Figure 3:**
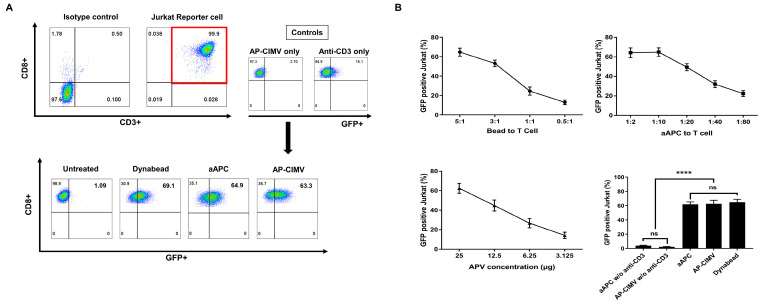
Activation of Jurkat reporter cells by aAPCs and AP-CIMVs coated with anti-CD3 antibody. (**A**) Induction of GFP expression in Jurkat reporter cells stimulated with aAPCs, AP-CIMVs, or Dynabead in the presence or absence of anti-CD3 antibody. Jurkat reporter cells were first gated with CD3+CD8+, and GFP expression was measured via flow cytometry. As background controls, Jurkat reporter cells cultured with AP-CIMVs alone or anti-CD3 antibody alone were measured. (**B**) Dose-dependent induction of GFP in Jurkat reporter cells stimulated with Dynabead, aAPCs, or AP-CIMVs. Representative data are shown. Every experiment was performed in triplicate and repeated at least twice. Error bars display mean values ± SD of triplicate. *p* value was calculated via one-way ANOVA. **** *p* < 0.0001.

GFP expression in Jurkat reporter cells was induced by stimulation with aAPC and AP-CIMVs coated with anti-CD3 antibody or anti-CD3/CD28 Dynabead. Jurkat reporter cells were first gated with CD3+CD8+ cells, and GFP expression was measured via flow cytometry (Figure 3A). GFP expression in Jurkat reporter cells was induced in a dose-dependent manner in all three groups. Anti-CD3/CD28 Dynabead at a bead:Jurkat reporter cell ratio of 5:1, aAPC at Jurkat reporter cell:aAPC ratio of 10:1, and AP-CIMV at 25 μg/1 × 10^5^ Jurkat reporter cells showed GFP expression of 69.1%, 64.9%, and 63.3%, respectively (Figure 3B). When Jurkat reporter cells were stimulated with AP-CIMVS alone or anti-CD3 antibody alone, GFP expression was not induced as much as in anti-CD3-coated AP-CIMVs. These results suggest that CD3 signaling using AP-CIMVs is comparable to that using aAPCs or anti-CD3/CD28 Dynabeads.

### 3.4. HLA-A*02:01 Restricted T Cell Activation by AP-CIMVs Loaded with CMV pp65 Peptide

**Figure 4:**
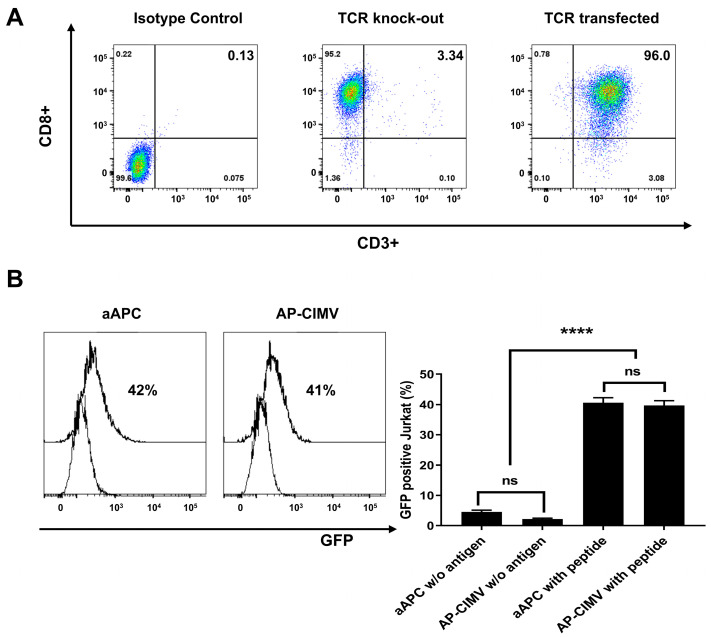
Activation of Jurkat reporter cells expressing CMV-specific TCR by aAPCs and AP-CIMVs expressing HLA-A*02:01 and loaded with CMV pp65 peptide. (NLVPMVATV) (**A**) In Jurkat cells in which the endogenous TCR was removed by Crispr/cas9, CD3 molecules do not appear on the cell surface. When mRNAs of HLA-A*02:01-restricted and CMV pp65-specific TCR alpha and beta genes were transferred, the reappearance of CD3 molecules on the cell surface indicated expression of the transferred TCR gene. (**B**) Induction of GFP expression in Jurkat reporter cells expressing specific TCR stimulated by aAPCs or AP-CIMVs expressing HLA-A*02:01 loaded with CMV pp65 epitope peptide in the negative control group, aAPC or AP-CIMVs alone without antigen peptide was used for stimulation. Error bars display mean values ± SD of triplicate. *p* value was calculated via one-way ANOVA. **** *p*< 0.0001.

To investigate T cell activation via HLA and peptide complexes, Jurkat reporter cells transduced with the CMV-specific TCR gene, aAPCs, and AP-CIMVs expressing HLA-A*02:01 were loaded with the CMV pp65 epitope peptide (NLVPMVATV) (Figure 4A). In Jurkat cells in which the endogenous TCR was removed via Crispr/cas9, CD3 molecules did not appear on the cell surface. When mRNAs of HLA-A*02:01-restricted and CMV pp65-specific TCR alpha and beta genes were transferred, the reappearance of CD3 molecules on the cell surface indicated the expression of the transferred TCR gene (Figure 4A). When Jurkat reporter cells were stimulated by aAPCs and AP-CIMVs loaded with CMV pp65 peptide, GFP expression by AP-CIMVs (41%) was similar to that by aAPCs (42%) (Figure 4B). When aAPCs and AP-CIMVs expressing HLA-B*07:02 were used to stimulate a Jurkat reporter cell line transfecting specific TCRs, similar results to those by HLA-A*0201 in Figure 4 were observed (Supplementary Material, Figure S1). In the negative control group, aAPC alone or AP-CIMVs alone without antigen peptide did not stimulate Jurkat reporter cells expressing a specific TCR. These results suggest that the HLA and peptide complexes presented by AP-CIMVs are similar to those of aAPCs.

### 3.5. Presentation of Endogenously Processed Antigen by AP-CIMVs

**Figure 5:**
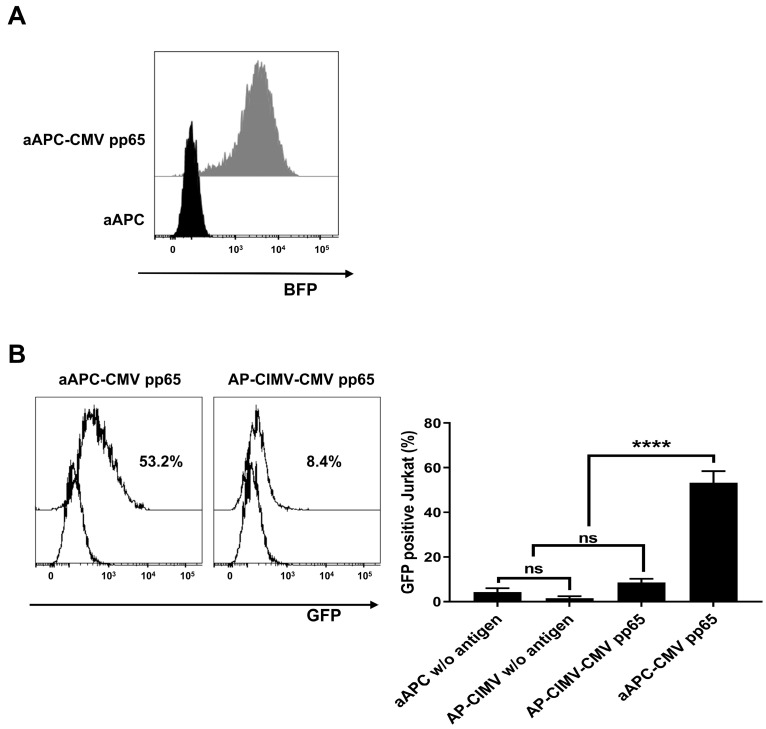
Activation of Jurkat reporter cells expressing specific TCR by aAPCs and AP-CIMVs presenting endogenously processed CMV pp65 antigen. (**A**) BFP co-expressed with CMV pp65 in aAPCs was analyzed via flow cytometry. (**B**) Jurkat reporter cells expressing specific TCR were stimulated with aAPCs expressing HLA-A*02:01 and CMV pp65 antigen (10:1) and their AP-CIMVs (25 ug), and then, the GFP expression was measured via flow cytometry. In the negative control group, aAPCs without antigen expression or their AP-CIMVs alone were used for stimulation. Representative data are presented. Error bars display mean values ±SD of triplicate. *p* value was calculated via one-way ANOVA. **** *p* < 0.0001.

To investigate whether HLA-expressing AP-CIMVs can effectively stimulate T cells by the presentation of endogenously processed antigens, we established aAPCs which stably express the CMV pp65 antigen (aAPC-CMV pp65). Antigen-specific T cell stimulation by the cells and their AP-CIMVs was measured (Figure 5A). The GFP expression of Jurkat reporter cells expressing specific TCR showed 53.2% by stimulation with aAPCs expressing CMV pp65 antigen; however, it was 8.4% by stimulation with their AP-CIMVs (Figure 5B). These data suggest that endogenously processed antigen-presenting AP-CIMVs have a limited effect on antigen-specific T cell stimulation compared to aAPCs. In order to increase the efficacy of T cell stimulation by AP-CIMVs, the mechanism of AP-CIMVs’ action should be more precisely elucidated.

## 4. Discussion

As cytochalasin B breaks down the form of the cell membrane by actin filament inhibition, the remaining cytochalasin B after CIMV separation may adversely affect recipient cells. However, CIMV is not toxic to recipient cells [6,7]. In this study, we observed the aggregation of CIMV during incubation to stimulate T cells in vitro or during the freezing and thawing process even with the standard isolation procedure for CIMV (Figure 1). This aggregation significantly reduced the recovery rate of CIMV when thawed after freezing. Furthermore, MV aggregation has been implicated in the formation of thrombosis or inflammatory disorders; thus, the risk is expected to be high when administered as a vaccine [19]. Because most of the remaining cells after treatment with cytochalasin B were found to be dead via trypan blue staining, DNA released from these cells was able to exist together within isolated CIMV and may cause this aggregation. A significant decrease in aggregation after nuclease treatment demonstrated this possibility (Figure 1). Furthermore, the recovery rate of nuclease-treated CIMV was not reduced, even after freezing and thawing. The isolation of CIMV without aggregation is important from a practical point of view for various applications of CIMV because it can be kept functionally stable by freezing [20].

In this study, the T-cell-stimulating ability of AP-CIMVs was measured with a method of anti-CD3 coating and antigen presentation with antigen pulsed or naturally processed. We used HLA-null HEK293T (H1E-45) cells, based on the HEK293T cell line for aAPCs [16]. A novel aAPC system expressing HLA and various co-stimulatory molecules efficiently stimulated T cells and enabled antigen-specific T cell proliferation in vitro [17]. AP-CIMVs isolated from these cells also expressed HLA and co-stimulatory molecules on their membranes (Figure 2). To evaluate the ability of AP-CIMVs to stimulate T cells, Jurkat reporter cells transfected with the human TCR specifically recognizing a complex of HLA-A*02:01 and a known CMV pp65 epitope peptide were used [21]. The most important benefit of the reporter cell line is that it can immediately measure NF-kB signaling activation through GFP expression on the cell surface without an additional staining process, making the assay simple, quick, and cost-effective [15]. The question of whether the response of Jurkat reporter cells can represent the response of primary human T cells has been previously investigated. In the study of primary T cells, AP-CIMVs induced a low T-cell-stimulatory response to the epitope peptide compared to that of aAPCs (Supplementary Material, Figure S2). Since the response to the epitope peptide was similar to AP-CIMVs and aAPCs when measured using Jurkat reporter cells, we found that Jurkat reporter cells were much more sensitive than primary T cells. Based on these observations, we decided to use sensitive Jurkat reporter cells in this study to precisely measure the ability of AP-CIMVs.

AP-CIMVs with anti-CD3 binding or peptide-sensitized HLA-A*02:01 exhibited a T cell stimulation effect similar to that of aAPCs, but AP-CIMVs which present naturally processed pp65 antigen by HLA-A*02:01 showed a very low T cell stimulation effect compared to that of aAPCs (Figure 3, Figure 4 and Figure 5). In our previous studies using exosomes, exosomes presenting naturally processed antigen by HLA-A*02:01 showed very low T cell stimulation effects compared to their original cells [13]. The rate at which a naturally processed antigen is presented by the HLA molecule may be very low compared with that of pulsing with a known epitope peptide [22]. Immunological synapses, known as supramolecular activation clusters (SMACs), are the interface between antigen-presenting cells and lymphocytes and consist of molecules involved in T cell activation which form a classic pattern. [23,24]. The radius of the immunological synapse is 5–10 μm, whereas that of CIMV is approximately 300–1100 nm [25]. The size of the aAPC or AP-CIMV is an important factor to initiate the signaling pathway because of the complex ratio that varies depending on the surface area. The smaller SMAC size of AP-CIMV means the antigen-specific antigen–HLA complex amount is less than it used to. However, it is presumed that the anti-CD3 antibody-coated AP-CIMVs stimulate T cells similarly to aAPCs because the number of TCRs that bind to them is sufficiently dense despite a small area. Therefore, in terms of the structure for stimulating T cells, the presentation of naturally processed antigen using CIMV may significantly lower the ability to activate T cells compared to that by aAPCs [26]. Even though CIMV lacks costimulatory molecules, the antigen-specific stimulation and expansion of CAR-T cells using CIMV-expressing CAR target molecules on the surface have been demonstrated to result in improved populations of functional CAR-T cells for therapy [14]. This CIMV was generated from HeLa cells expressing target molecules, such as CD19, IL-13R2A, CD33, CD123, and HER2. [14]. As the domains of CD3z and co-stimulatory molecules are included in the signaling domain of CAR, they may differ from antigen presentation by HLA molecules of CIMV, in which the co-stimulatory molecules must express separately [27,28].

## 5. Conclusions

In this study, to investigate the possibility of AP-CIMVs expressing HLA molecules as a cell-free system capable of activating T cells, the ability to stimulate T cells under various antigen presentation conditions using CIMVs was precisely measured with a Jurkat reporter cell line. When stimulated with anti-CD3 antibody or epitope peptide, AP-CIMVs showed comparable T-cell-stimulatory functions to aAPCs, but they were significantly lowered when stimulated with naturally processed antigen. These results prove the limitations of AP-CIMVs for the presentation of naturally processed antigens, suggesting that further studies are needed to improve their functions.

## Data Availability

The data presented in this study are available within the article.

## Supplementary Materials

The following supporting information can be downloaded at: https://www.mdpi.com/article/10.3390/vaccines10111877/s1, Figure S1: Activation of Jurkat reporter cells expressing CMV-specific TCR by aAPCs and AP-CIMVs expressing HLA-B*07:02 and loaded with CMV pp65 peptide (RPHERNGFTVL); Figure S2: Activation of naïve human CD8+T cells using CMV pp65 peptide coated aAPCs and AP-CIMVs.
