# Supplementary material for: Limited T-Cell-Stimulating Effect of Cytochalasin-B-Induced Membrane Vesicles Isolated from Artificial Antigen-Presenting Cells
Source: Vaccines (Basel). 2022 Nov 7;10(11):1877. doi: 10.3390/vaccines10111877 (PMC9694503; doi:10.3390/vaccines10111877)
Supplement: Supplementary file 1 [file vaccines-10-01877-s001.zip › vaccines-1977657-supplementary.pdf]

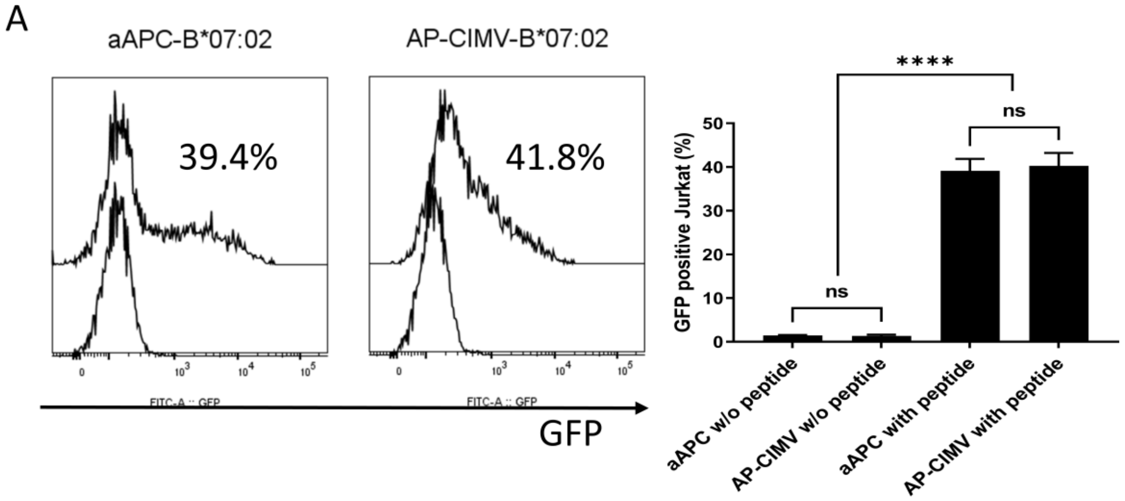

Figure S1. Activation of Jurkat reporter cells expressing CMV-specific TCR by aAPCs and AP-CIMVs expressing HLA-B*07:02 and loaded with CMV pp65 peptide (RPHERNGFTVL). (**A**) Induction of GFP expression in Jurkat reporter cells expressing specific TCR stimulated by aAPC or AP-CIMVs expressing HLA-B*07:02 loaded with CMV pp65 epitope peptide in the negative control group, aAPC or AP-CIMVs alone without antigen peptide was used to stimulate. Error bars display mean values ±SD of triplicate. *P* value was calculated by one-way ANOVA. **** $p < 0.0001$.

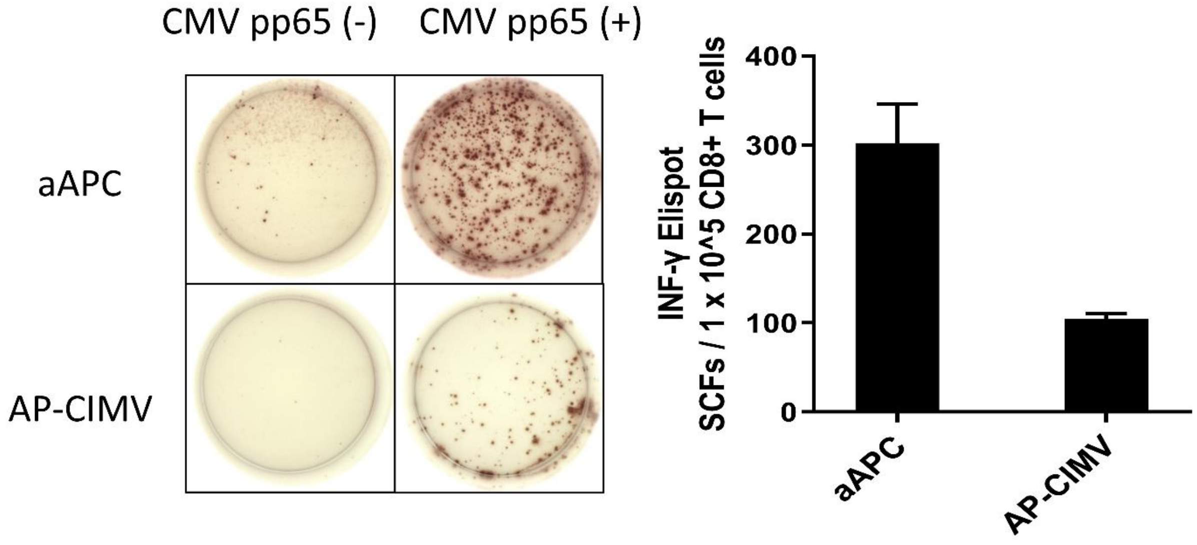

Figure S2. Activation of naïve human CD8+T cells using CMV pp65 peptide coated aAPCs and AP-CIMVs. CMV pp65 specific T cell stimulation was measured by secreted IFN-γ capture on antibody coated ELISPOT culture plate and analyzed. (**A**) The representative ELISPOT images of healthy donor using aAPC or AP-CIMV with or without peptide pulsing. (**B**) The result of IFN-γ ELISPOT assay of three healthy donors expressing HLA-A*02:01. Spot forming cells (SFCs) were counted using an AID ELISPOT Reader System (AID Diagnostika GmbH) and subtracted the background spot numbers. Error bars display mean values ±SD of triplicate.
